# Autochthonous Case of Pulmonary Histoplasmosis, Switzerland 

**DOI:** 10.3201/eid2703.191831

**Published:** 2021-03

**Authors:** Yvonne Schmiedel, Annina E. Büchi, Sabina Berezowska, Alexander Pöllinger, Konrad Mühlethaler, Manuela Funke-Chambour

**Affiliations:** Basel University Hospital, Basel, Switzerland (Y. Schmiedel);; Hôpital du Jura, Delémont, Switzerland (Y. Schmiedel);; Inselspital, Bern University Hospital, University of Bern, Bern, Switzerland (Y. Schmiedel, A.E. Büchi, A. Pöllinger, M. Funke-Chambour);; Lausanne University Hospital and University of Lausanne, Lausanne, Switzerland (S. Berezowska);; Institute of Pathology, University of Bern, Bern, Switzerland (S. Berezowska);; Institute for Infectious Diseases, University of Bern, Bern (K. Mühlethaler)

**Keywords:** fungi, Histoplasma capsulatum, histoplasmosis, pulmonary histoplasmosis, respiratory infections, Switzerland

## Abstract

In Europe, pulmonary histoplasmosis is rarely diagnosed except in travelers. We report a probable autochthonous case of severe chronic pulmonary histoplasmosis in an immunocompetent man in Switzerland without travel history outside of Europe. Diagnosis was achieved by histopathology, fungal culture, and serology, but the source of the infection remains speculative.

A 48-year-old man in Switzerland sought treatment for a 1-year history of progressive dyspnea, cough, 20-kg weight loss, and increased sweating; he was receiving oxygen therapy. Results of previous consultations had been inconclusive. An HIV screening test was negative. Medical history included hyperreflexia, depression, and chronic hepatitis B. The man had stopped cocaine inhalation and heroin consumption 20 years earlier but continued smoking cigarettes and cannabis. Regular medications included omeprazole and trimipramine. Except for a short trip to Greece and Italy many years before, the patient reported no foreign travel. 

In the absence of travel history to an endemic area, histoplasmosis was not initially considered at the time this patient sought treatment. A prolonged diagnostic process and delayed treatment initiation had meanwhile resulted in significant deterioration of health, including need for oxygen therapy, and loss of ability to work. Meanwhile, the patient was cachectic and had clubbing on his fingers and toes. Spirometry revealed nearly normal dynamic lung volumes. Forced expiratory volume was 3 L (75%) and forced vital capacity 4.1 L (83%), but diffusion capacity was severely impaired; diffusing capacity for carbon monoxide was 20%. A 6-minute walking test was limited to 400 m (59% predicted), initial oxygen saturation dropping from 90% to 78%. A chest computed tomography (CT) scan showed a diffuse reticulonodular pattern with predominantly upper lung opacifications and bronchiectases indicating fibrotic lung disease (Figure, panels A, B). Reversed halo signs and right upper lobe nodules were found. Bronchoscopy results including bronchoalveolar lavage were unremarkable. Initial sampling with microbiological screening was negative. 

**Figure:**
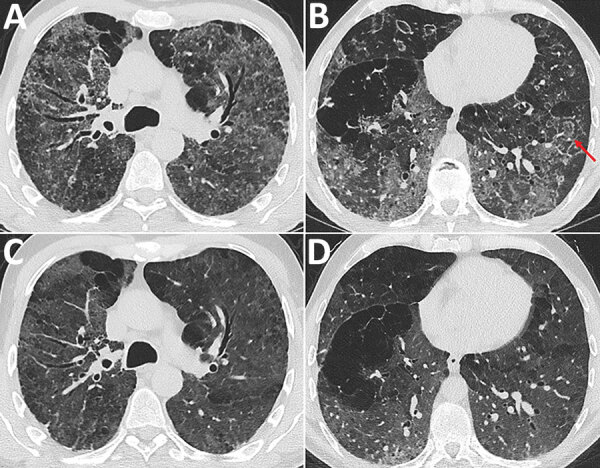
Chest computed tomography (CT) images at the level of the upper third and the lower third of the lung in a patient with pulmonary histoplasmosis, Switzerland. A, B) Initial CT shows diffuse reticulonodular pattern with ground glass opacifications, predominantly located in the upper two thirds of the lungs, and several areas with reverse halo signs (red arrows). C, D) Follow-up CT scan exhibited reduced ground-glass opacities and a regression of the micronodules. The reversed halos showed complete regression. CT, computed tomography.

Differential diagnoses included toxic lung damage or other interstitial lung disease, (e.g. atypical presentation of Langerhans cell histiocytosis or sarcoidosis). A wedge biopsy showed predominantly upper-lobe fibrosis and multiple, confluent, necrotizing granulomas harboring yeasts, establishing the diagnosis of pulmonary histoplasmosis (Appendix Figure). 

A qualitative immunodiffusion test (IMMY, https://www.immy.com) was positive for antibodies in plasma, but an antigen immunoassay for *Histoplasma* in urine (IMMY) was negative; a beta-1,3-D glucan test (Fungitell, https://www.fungitell.com) was highly positive (>500 pg/mL; limit <80 pg/mL). At prolonged incubation (14 days, 30°C), a fungal culture on BD Difco dehydrated culture media Sabouraud brain heart infusion agar base (with chloramphenicol and cycloheximide) (https://www.bd.com) showed flat, floccose to powdery, whitish growth. We found microscopically large, tuberculated macroconidia (7–12 µM) and small round microconidia on short, lateral pegs consistent with *Histoplasma capsulatum*. Matrix-assisted laser desorption/ionization time-of-flight mass spectrometry (MALDI Biotyper, https://www.bruker.com) results confirmed the diagnosis. Molecular identification was done using an in-house panfungal PCR assay with consecutive sequence analysis. We used the internal transcribed spacer region as target and internal transcribe sequences 1 and 2 for amplification primers (*1*,*2*). Microsynth AG (https://www.microsynth.ch) performed DNA sequencing. Sequences produced alignments of *H. capsulatum* in BLAST (https://blast.ncbi.nlm.nih.gov/Blast.cgi) and CBS (Centraalbureau voor Schimmelcultures; Westerdijk Institute, https://wi.knaw.nl) databases. 

Some radiologic features were unusual. There was no cavity formation (*3*), and the reverse halo sign has rarely been described in chronic pulmonary histoplasmosis (*4*). However, bullae seen on the scan, previously observed in patients with heavy tobacco use and underlying lung disease, were compatible with the diagnosis. Despite slow growth, cultures for histoplasmosis together with histopathology remain the diagnostic standard (*1*). Panfungal PCR is sensitive, but its performance depends on internal validation processes (*2*). Immunocompetence and lack of dissemination could explain repeatedly negative urine antigen testing. (*1*).

Underlying lung disease likely predisposed this patient for severe disease. However, his clinical response to treatment was remarkable. We initiated antifungal treatment with liposomal amphotericin B and oral prednisolone. After a few days, the patient improved substantially, and oxygen supplementation was stopped. At 10 days, therapy was switched to oral itraconazole. Steroid treatment was continued at a tapered dosage over 3 months, with trimethoprim/sulfamethoxazole used as *Pneumocystis jirovecii* pneumonia prophylaxis. At 3-month follow-up, the patient had improved considerably. Repeated spirometry was nearly normal, showing persistent impairment of diffusion capacity. Follow-up chest CT scan (Figure, panels C, D) showed regression of ground-glass opacities and micronodules; the reversed halos had disappeared. Overall, optimal treatment duration remains unclear (*5*), but because of probable underlying preexisting lung disease, persistent pathological findings from CT, and continued desaturation under exercise, continuing treatment for >12 months seemed necessary.

The source of infection for this patient remains speculative. However, possible risk exposures were guano from flying bats in the garden (*6*), previous use of organic fertilizer possibly containing histoplasma (*7*), and regular work-related unpacking of fruits and spices from straw-filled boxes from West Africa, although *H. capsulatum* var. *capsulatum* is less common in that region (*8*). 

In addition to previous findings of histoplasmosis in badgers (*9*), this case confirms the likely environmental occurrence of *H. capsulatum* in Switzerland. Although diagnoses of autochthonous histoplasmosis have been rare, and few autochthonous cases have been described (*10*), our finding of a probable autochthonous case of chronic pulmonary histoplasmosis in an immunocompetent male in Switzerland highlights the incomplete understanding of histoplasmosis endemicity and indicates that it has likely been underestimated in Europe. 

Appendix Additional information on an autochthonous case of pulmonary histoplasmosis in Switzerland.
