# Supplementary material for: Autochthonous Case of Pulmonary Histoplasmosis, Switzerland
Source: Emerg Infect Dis. 2021 Mar;27(3):966–9. doi: 10.3201/eid2703.191831 (PMC7920691; doi:10.3201/eid2703.191831)
Supplement: Appendix — Additional information on an autochthonous case of pulmonary histoplasmosis in Switzerland. [file 19-1831-Techapp-s1.pdf]

Article DOI: https://doi.org/10.3201/eid2703.191831

Autochthonous Case of Pulmonary Histoplasmosis, Switzerland

Appendix

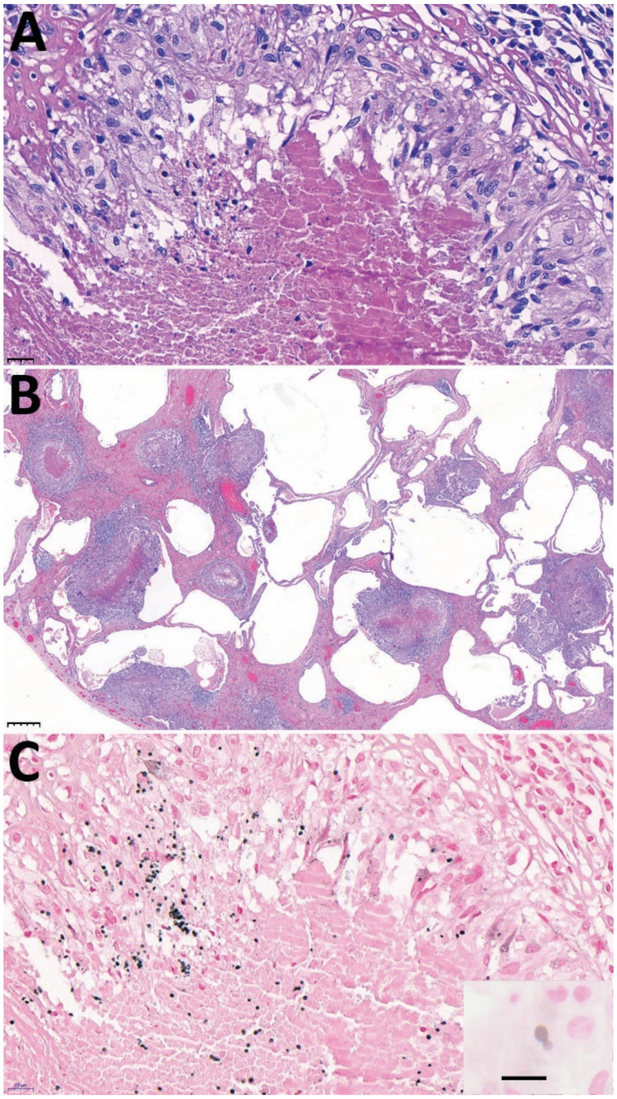

Appendix Figure. Diagnostic wedge-biopsy establishing diagnosis of pulmonary histoplasmosis. A) (hematoxylin and eosin staining; 20× magnification) Overview of the diagnostic wedge-biopsy showing emphysema, fibrosis, and necrotizing granulomas. B) Higher magnification (hematoxylin and eosin staining; 400×) highlights the necrosis and the surrounding histiocytic wall. C) (400× magnification) Grocott silver staining discloses the ovoid histoplasma organisms (black staining).
